# A multicentric evaluation of the recombinant *Leishmania infantum* antigen-based immunochromatographic assay for the serodiagnosis of canine visceral leishmaniasis

**DOI:** 10.1186/1756-3305-7-136

**Published:** 2014-03-31

**Authors:** Deborah Bittencourt Mothé Fraga, Edimilson Domingos da Silva, Luciano Vasconcellos Pacheco, Lairton Souza Borja, Isaac Queiroz de Oliveira, Wendel Coura-Vital, Glória Regina Monteiro, Geraldo Gileno de Sá Oliveira, Selma Maria Bezerra Jerônimo, Alexandre Barbosa Reis, Patrícia Sampaio Tavares Veras

**Affiliations:** 1 Laboratório de Patologia e Biointervenção, Centro de Pesquisa Gonçalo Moniz, FIOCRUZ, Salvador, Bahia, Brazil; 2 Departamento de Medicina Veterinária Preventiva e Produção Animal, Escola de Medicina Veterinária e Zootecnia, Universidade Federal da Bahia, Salvador, Bahia, Brazil; 3 Instituto de Ciência e Tecnologia de Doenças Tropicais, INCT-DT, Bahia, Brazil; 4 Laboratório de Tecnologia Diagnóstica, Instituto de Tecnologia em Imunobiológicos, Bio-Manguinhos, FIOCRUZ, Rio de Janeiro, Rio de Janeiro, Brazil; 5 Pós-graduação em Infectologia e Medicina Tropical, Faculdade de Medicina, Universidade Federal de Minas Gerais, Belo Horizonte, Minas Gerais, Brazil; 6 Laboratório de Imunopatologia, Núcleo de Pesquisas em Ciências Biológicas, Universidade Federal de Ouro Preto, Ouro Preto, Minas Gerais, Brazil; 7 Departamento de Bioquimica, Centro de Biociências, Universidade Federal do Rio Grande do Norte, Natal, Rio Grande do Norte, Brazil

**Keywords:** Canine visceral leishmaniasis, Serological diagnosis, Recombinant antigen, Rapid test, Multicentric study

## Abstract

**Background:**

Visceral leishmaniasis (VL) is a serious public health challenge in Brazil and dogs are considered to be the main urban reservoir of the causative agent. The culling of animals to control VL in some countries makes the accurate diagnosis of canine VL (CVL) essential. Recombinant antigens rLci1A and rLci2B were selected from a cDNA library of *Leishmania infantum* amastigotes due to their strong potential as candidates in diagnostic testing for CVL. The present multicentric study aimed to evaluate the sensitivity of a prototype test using these antigens (DPP rLci1A/rLci2B) against 154 sera obtained from symptomatic dogs within three endemic areas of VL in Brazil. The specificity was evaluated using 40 serum samples from negative dogs and dogs infected with other pathogens. Sensitivity and specificity rates of DPP rLci1A/rLci2B prototype were compared to rates from other diagnostic tests currently in use by the Brazilian Ministry of Health, including DPP®LVC, EIE®LVC.

**Findings:**

DPP rLci1A/rLci2B prototype offered similar performance to that offered by DPP®LVC rapid test, as follows: sensitivity of 87% (CI 81–91) and 88% (CI 82–93) and specificity of 100% (CI 91–100) and 97% (CI 87–100), respectively for DPP rLci1A/rLci2B and DPP®LVC. When results of these two tests were considered concomitantly, sensitivity increased to 93.5% (CI 89–96).

**Conclusions:**

The recombinant antigens rLci1A and rLci2B represent promising candidates for use in a multi-antigen rapid test for CVL. The inclusion of novel antigens to the DPP rLci1A/rLci2B prototype model could offer additionally enhanced sensitivity to detect animals infected by *L. infantum*.

## Findings

Visceral leishmaniasis (VL) is caused by infection with *Leishmania infantum* and dogs are considered to be the main urban reservoir. Some countries advocate the culling of dogs infected with *L. infantum*[1] as a control measure for VL, making accurate diagnosis crucial in order to correctly identify animals infected with *L. infantum*[2,3]. Until December 2011, the Brazilian Ministry of Health recommended the use of enzyme-linked immunosorbent assays (ELISA) and indirect immunofluorescence assays (IFA) to diagnose *L. infantum* infection in dogs [4]. Unfortunately, these immunological assays, offer moderate sensitivity and specificity, thereby contributing to the maintenance of infected animals in endemic areas [5,6]. An immunochromatographic rapid test (DPP®LVC) based on the rK28 has recently become the preferred diagnostic method for screening in Brazil, followed by ELISA (EIE®LVC) as a confirmatory test. A recent study demonstrated 98% sensitivity using DPP®LVC in symptomatic dogs, yet found low sensitivity (47%) in asymptomatic dogs [7]. Putting the use of this protocol for canine visceral leishmaniasis (CVL) diagnosis under scrutiny. For improving VL control measures, the identification of novel recombinant antigens may contribute to enhance test sensitivity.

The antigens used in the prototype test evaluated in this study, rLci1A and rLci2B, were selected from a cDNA library of *L. infantum* amastigotes due to their reactivity to antibodies from naturally infected dogs [8]. A previous study demonstrated that rLci1A and rLci2B offer 96% and 100% sensitivity, with respective specificity rates of 92% and 95% under ELISA against sera from animals with positive parasitological test results [9]. These findings clearly indicate the potential of these selected antigens for use in CVL diagnosis.

The present study aimed to evaluate the sensitivity of antigens rLci1A and rLci2B impregnated in an immunochromatographic rapid test prototype based on the dual path platform—DPP (hereafter referred to as DPP rLci1A/rLci2B) for the serodiagnosis of dogs infected by *L. infantum* in three endemic areas of VL. Moreover, test sensitivity and specificity was compared to the DPP®LVC and EIE®LVC tests, which are actively used to diagnose CVL in endemic regions of Brazil.

## Methods

### Study design

The present multicentric study aimed to evaluate the performance offered by the DPP rLci1A/rLci2B prototype test for the serodiagnosis of CVL. A total of 154 serum samples were obtained from naturally infected symptomatic dogs in three endemic areas of Brazil, which presented evidence of active *L. infantum* infection in culture. The included sera were provided by the serum banks of three laboratories of The National Institute of Science and Technology in Tropical Diseases (INCT-DT), located in Salvador–Bahia (BA) (n = 53), Natal–Rio Grande do Norte (RN) (n = 50) and Ouro Preto–Minas Gerais (MG) (n = 51). Animal population from RN is formed by domiciled dogs, from MG by stray dogs and from BA by both domiciled and stray dogs. All of 154 dogs presented more than 3 signs at clinical examination. *L. infantum* infection was identified using multilocus enzyme electrophoresis of parasites isolated from cultures of splenic aspirates taken from dogs from RN and BA, and using PCR-RFLP of parasites isolated from cultures of splenic or bone marrow aspirates from dogs from MG. A total of 40 serum samples, 20 from negative dogs, 5 infected by *Leishmania braziliensis*, 5 by *Trypanosoma cruzi*, 6 by *Ehrlichia canis* and 4 by *Babesia canis*, selected from a serum bank located in BA, were used to evaluate the specificity offered by the tests evaluated: DPP®LVC, EIE®LVC and DPP rLci1A/rLci2B prototype test.

### Antigen production and purification

*Escherichia coli* BL21(DE3)pLysS (Invitrogen) were transformed with pRSET plasmids (Invitrogen) containing the Lci1A or Lci2B *L. infantum* gene insert. Affinity chromatography was used to purify the rLci1A and rLci2B proteins as previously described [9].

### Prototype production

The DPP rLci1A/rLci2B prototype test employed rLci1A and rLci2B antigens impregnated on nitrocellulose membrane strips in individual bands. This prototype utilizes the same platform as the DPP®LVC (Biomanguinhos).

Two prototype models were produced, each with different concentrations of the two antigens: 1) rLci1A and rLci2B at 0.35 mg/mL and 0.20 mg/mL; 2) rLci1A and rLci2B at 0.70 mg/mL and 0.70 mg/mL.

### Diagnostic test procedures

Each of the three included INCT-DT laboratories conducted DPP rLci1A/rLci2B prototype diagnostic test procedures in accordance with manufacturer recommendations (BioManguinhos/FIOCRUZ) and the visual result was interpreted. If a band was visible in the test area, likewise in the control area, the result was considered positive. When no bands were visible in the test area, but one was present in the control area, a negative result was recorded. All other band combinations were disregarded.

### Analysis of prototype test results

Sensitivity and specificity rates were calculated with 95% confidence interval. Sensitivity and specificity rates of the DPP®LVC and EIE®LVC tests were compared to sensitivity of the DPP rLci1A/rLci2B prototype. Kappa index [10] was calculated by STATA program (StataCorp LP, College Station, Texas, USA) as an agreement measure among tests with respect to a given sample. Fisher’s exact test was used to identify differences among positivity rates from diagnostic tests using GraphPad Prism software (Graph Pad, San Diego, California, USA). *p* value less than 0.05 was considered significant.

### Ethical considerations

All serological sample obtainment procedures employed were submitted for approval by the Institutional Review Board for Animal Research at the Gonçalo Moniz Research Center in Salvador, Bahia, in addition to the institutional review boards of the other included centers.

## Results and discussion

DPP®LVC and EIE®LVC test sensitivity remained largely consistent among centers (Table 1), with no statistically significant differences detected. DPP®LVC provided 92.5% sensitivity in sera from Bahia (BA), 84% in sera from Minas Gerais (MG) and 88.2% in samples from Rio Grande do Norte (RN). The EIE®LVC test offered the highest sensitivity of all the techniques evaluated, with 98% in sera from BA, 93.5% in samples from MG and 100% in sera from RN.

**Table 1:** Comparison of diagnostic test sensitivity

**Serum sample**	**Comparison of diagnostic test sensitivity (95% CI) [n/N]**					
**Source**	**EIE®LVC**	**DPP® LVC**	**DPP rLci1A**	**DPP rLci2B**	**DPP rLci1A/rLci2B**	**DPP® LVC + DPPrLci1A/rLci2B**
BA	98%	92.5%	66%	81.1%	83%	96.2%
	(86–100)	(82–97)	(53–77)	(69–89)	(71–91)	(87–99)
	[49/50]	[49/53]	[35/53]	[43/53]	[44/53]	[51/53]
MG	93.5%	84%	70%	76%	84%	88%
	(92–100)	(72–92)	(56–81)	(63–86)	(72–92)	(76–94)
	[43/46]	[42/50]	[35/50]	[38/50]	[42/50]	[44/50]
RN	100%	88.2%	90.2%	90.2%	94.1%	96%
	(93–100)	(77–95)	(79–96)	(79–96)	(84–98)	(87–99)
	[51/51]	[45/51]	[46/51]	[46/51]	[48/51]	[49/51]
Total*	97.3%	88.3%	75.3%	82.5%	87%	93.5%
	(93–99)	(82–93)	(68–82)	(76–88)	(81–91)	(89–96)
	[143/147**]	[136/154]	[116/154]	[127/154]	[134/154]	[144/154]

*Abbreviations*: CI = confidence interval, n = number of sera that tested positive, N = number of sera evaluated.

*154 canine serum samples tested positive for VL in culture and were obtained from three participating INCT-DT laboratories.

**7 out of 154 canine serum samples were not considered under ELISA due to inconclusive test results.

**Figure 1:**
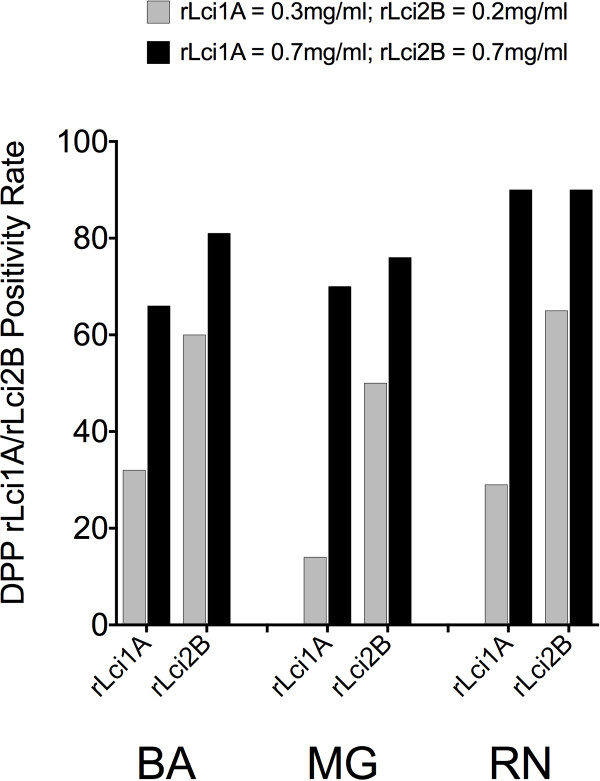
**Positivity rates of DPP rLci1A / rLci2B regarding detection of serum samples from *L. infantum*-infected dogs.** The antigens rLci1A / rLci2B were individually impregnated at concentrations of 0.35/0.20 mg/mL and 0.70/0.70 mg/mL, respectively. Positivity rates for either proteins were analyzed both individually and collectively. A total of 154 canine serum samples were obtained from the three INCT-DT laboratories included in this study.

To evaluate the sensitivity of the DPP rLci1A/rLci2B prototype, the 154 canine sera included were initially analyzed by considering reactivity to each recombinant antigen separately. Under lower antigen concentrations, rLci1A (0.35 mg/mL) was detected in 32% of the sera from BA, 14% from MG and 29% from RN. Notably, at the higher concentration (0.70 mg/mL), rLci1A sensitivity increased to 66%, 70% and 90%, respectively. Similarly, when rLci2B was impregnated at a lower concentration (0.20 mg/mL), only 60% of the sera from BA, 50% from MG and 65% from RN tested positive. However, at the higher concentration of 0.70 mg/mL, rLci2B sensitivity increased considerably to 81%, 76% and 90%, respectively (Figure 1, Table 1).

Next, the global test results of the DPP rLci1A/rLci2B prototype model were evaluated by considering the resulting sensitivity when at least one of the two proteins evaluated were detected by antibodies in serum samples at higher concentrations (0.70 mg/mL). The observed sensitivity of the dual-antigen prototype test increased considerably, with 83% of the sera from BA, 84% from MG and 94% from RN presenting reactivity to at least one of the two antigens present in the prototype test (Table 1). The corresponding Kappa index value of 0.5162 indicated moderate agreement with respect to rLci1A and rLci2B recognition among the 154 samples, suggesting that sensitivity increases by up to 12% under a dual-antigen prototype.

The DPP®LVC and DPP rLci1A/rLci2B tests exhibited remarkably similar rates of sensitivity: 88% and 87%, respectively. Agreement between these two DPP test formats was moderate, with a Kappa index value of 0.459 (P < 0.001).

A lack of agreement was observed in 18 out of 154 sera when comparing DPP®LVC and DPP rLci1A/rLci2B results. Ten samples tested positive under DPP®LVC and negative using the DPP rLci1A/rLci2B prototype, while eight were negative under DPP®LVC yet positive using the prototype.

Since sera from asymptomatic dogs were not included in the present report, the accuracy rates obtained herein may be overestimated.

The specificity of the tests is shown in Table 2. Briefly, the observed specificity values were 97% for DPP® LVC and 100% for the DPP rLci1A/rLci2B prototype, values that were much higher than that for EIE®LVC (26%). It is noteworthy that EIE®LVC offered the highest sensitivity rate among all techniques evaluated, although it presented the lowest specificity value.

**Table 2:** Comparison of diagnostic test specificity

**Serum sample**	**Comparison of diagnostic test specificity (CI) [n/N]**				
	**EIE®LVC**	**DPP® LVC**	**DPP rLci1A**	**DPP rLci2B**	**DPP rLci1A/rLci2B**
*L. braziliensis*	25%	100%	100%	100%	100%
	(5–70)	(57–100)	(57–100)	(57–100)	(57–100)
	[3/4]	[0/5]	[0/5]	[0/5]	[0/5]
*T. cruzi*	50%	100%	100%	100%	100%
	(15–85)	(57–100)	(57–100)	(57–100)	(57–100)
	[2/4]	[0/5]	[0/5]	[0/5]	[0/5]
*E. canis*	67%	100%	100%	100%	100%
	(30–90)	(61–100)	(61–100)	(61–100)	(61–100)
	[2/6]	[0/6]	[0/6]	[0/6]	[0/6]
*B. canis*	50%	100%	100%	100%	100%
	(15–85)	(51–100)	(51–100)	(51–100)	(51–100)
	[2/4]	[0/4]	[0/4]	[0/4]	[0/4]
Negative dogs	88%	95%	100%	100%	100%
	(66–97)	(76–99)	(84–100)	(84-100	(84–100)
	[2/17]	[1/20]	[0/20]	[0/20]	[0/20]
Total	26%	97.5%	100%	100%	100%
	(14–42)	(87–100)	(91–100)	(91–100)	(91–100)
	[26/35]	[1/40]	[0/40]	[0/40]	[0/40]

*Abbreviations*: C = confidence interval, n = number of sera that tested positive, N = number of sera evaluated.

Taken together, the results presented herein demonstrate that a multi-antigen DPP test format would enhance sensitivity, as evidenced by an up to 5% increase in test sensitivity using a prototype model employing three antigens. The findings herein are further supported by previous studies utilizing recombinant antigens from different pathogens, which found similarly favourable results [11-14]. The rationale for using more than one recombinant antigen is based on a study by Teixeira *et al*. [15], who demonstrated that the concomitant use of recombinant antigens can increase diagnostic test sensitivity. This finding can be explained by the production of a unique set of antibodies against a specific set of parasite antigens by each individual who becomes infected with *Leishmania*.

In conclusion, rLci1A and rLci2B represent promising candidates for inclusion in a future version of the DPP rapid test for the serodiagnosis of CVL. Preliminary results indicate that by combining these two antigens together with the existing rK28 antigen DPP®LVC rapid test currently in use, sensitivity would increase from 88% to 93.5%. Further investigation is warranted to comprehensively determine the efficacy of a multi-antigen test in field applications.

## Abbreviations

CI: Confidence interval; CVL: Canine visceral leishmaniasis; DPP: Dual path platform.

## Competing interests

The authors declare that they have no competing interests.

## Authors’ contributions

DBMF performed DPP®LVC and data analysis and wrote the manuscript. EDS prepared the DPP rLci1A/rLci2B prototypes. LVP, LSB and IQO organized the canine samples from BA laboratory and performed DPP®LVC, DPP rLci1A/rLci2B e EIE®LVC analysis, performed data analysis and tabulation. GGSO, SMBJ and ABR participated in the design of the multicentric study and revision of the manuscript. PSTV conceived the study, participated in its design and coordination and helped to draft the manuscript. WCV and GRM organized the canine samples from MG and RN laboratories and performed DPP rLci1A/rLci2B analysis. All authors read and approved the final manuscript.
